# A Machine Learning Approach for Identifying Gene Biomarkers Guiding the Treatment of Breast Cancer

**DOI:** 10.3389/fgene.2019.00256

**Published:** 2019-03-27

**Authors:** Ashraf Abou Tabl, Abedalrhman Alkhateeb, Waguih ElMaraghy, Luis Rueda, Alioune Ngom

**Affiliations:** ^1^Department of Mechanical, Automotive and Materials Engineering, University of Windsor, Windsor, ON, Canada; ^2^School of Computer Science, University of Windsor, Windsor, ON, Canada

**Keywords:** breast cancer, classification, feature selection, gene biomarkers, machine learning, cancer survivability, treatment therapy

## Abstract

Genomic profiles among different breast cancer survivors who received similar treatment may provide clues about the key biological processes involved in the cells and finding the right treatment. More specifically, such profiling may help personalize the treatment based on the patients’ gene expression. In this paper, we present a hierarchical machine learning system that predicts the 5-year survivability of the patients who underwent though specific therapy; The classes are built on the combination of two parts that are the survivability information and the given therapy. For the survivability information part, it defines whether the patient survives the 5-years interval or deceased. While the therapy part denotes the therapy has been taken during that interval, which includes hormone therapy, radiotherapy, or surgery, which totally forms six classes. The Model classifies one class vs. the rest at each node, which makes the tree-based model creates five nodes. The model is trained using a set of standard classifiers based on a comprehensive study dataset that includes genomic profiles and clinical information of 347 patients. A combination of feature selection methods and a prediction method are applied on each node to identify the genes that can predict the class at that node, the identified genes for each class may serve as potential biomarkers to the class’s treatment for better survivability. The results show that the model identifies the classes with high-performance measurements. An exhaustive analysis based on relevant literature shows that some of the potential biomarkers are strongly related to breast cancer survivability and cancer in general.

## Introduction

Despite the fast increase in the breast cancer incidence rate, the survival rates have also increased due to improvements in the treatments because of new technologies (Siegel et al., 2016). Breast cancer, however, is still one of the leading causes of cancer-related death among women worldwide. The survival rates vary among the various treatment therapies that are currently used, which include surgery, chemotherapy, hormone therapy, and radiotherapy. Nevertheless, each patient’s response to a specific treatment varies based on some factors that are being investigated (Miller et al., 2016).

Traditional laboratory techniques like CAT scans and magnetic resonance imaging (MRI) have been proven to be useful. However, they provide very little information about the mechanism of the cancer progression. On the contrary, advances in DNA microarray technology have provided high throughput samples of gene expression. Machine learning approaches have been utilized to detect breast cancer treatment or survivals (Mangasarian and Wolberg, 2000; Cardoso et al., 2016; Abou Tabl et al., 2017; Tang et al., 2017; Zeng et al., 2018). many researchers have used DNA microarray technology to study breast cancer survivability (Mangasarian and Wolberg, 2000; Cardoso et al., 2016; Abou Tabl et al., 2017). Analyzing gene expression among breast cancer patients who undergo varying treatment types deepens the current understanding of the disease’s progression and prognosis. Many features complicate the computational model; the number of features is usually significantly larger than the number of samples, which is known as the curse of dimensionality problem, in which standard classifiers overfit the data, and hence, perform poorly. Therefore, feature selection techniques are proven to alleviate the curse of dimensionality by removing irrelevant and/or redundant features.

Zou et al. (2016) proposed maximum Relevance maximum distance feature selection approach mRMD 2.0. The method uses Pearson’s correlation coefficient to measure the Relevance between sub group of features and the class. The selection criteria balance the accuracy with stability when selecting the features. The authors compared the dimensionality reduction method with both filter and wrapper feature selection types, and the results show that mRMD 2.0 outperformed different features selection method of each type (Zou et al., 2016). We compared mRMD 2.0 with mRMR on the wrapper phase of feature selection, while the accuracy of random forest on the selected features of each method was very close, mRMR overall selected less number of potential biomarkers with 47 genes compared to 60 genes of the mRMD 2.0, Hence, we utilized mRMR in this model to obtain a handful smaller size of potential biomarkers for further analysis.

Tang et al. (2017) predicted a tumor location in breast tissue based on feature selection method where the features are RNA-Seq and miRNA data, they enhanced the prediction of the standard classifiers to be around 93% in average. While Zeng et al. (2018) investigated a potential miRNA biomarker for breast neoplasm with around 80% accuracy. Mangasarian and Wolberg (2000) utilized a linear support vector machine (SVM) to extract 6 out of 31 clinical features. Their dataset contains samples from 253 breast cancer patients. The model involved classifying the samples into two groups: (1) the node-positive group in which the patients have some metastasized lymph nodes, and (2) the node-negative group for patients with no metastasized lymph nodes. Those six features were then used in a Gaussian SVM classifier to classify patients into three prognostic groups: negative, middle, or positive. The researchers found that patients in the negative group had the highest survivability. Most of these patients had received chemotherapy treatment (Mangasarian and Wolberg, 2000).

Using samples from patients with high-risk clinical features in the early stages of breast cancer, Cardoso et al. (2016) proposed the use of a statistical model to determine the necessity of chemotherapy treatment based on clinical data. In one of our earlier works, we built a prediction model based on various treatments without defining the period of survivability (Abou Tabl et al., 2017); that is, given a training dataset consisting of gene expression data of BC patients who survived or died after receiving a treatment therapy, we built a classification model that is used to predict whether a new patient will survive or die. In another work, we have implemented an unsupervised learning approach to find the separation between the treatment-survival groups of classes (Tabl et al., 2018a), the model is grouping different classes together in building the tree model while defining the border between the different groups of classes. Paredes-Aracil et al. (2017) built a scoring prediction system for 5 and 10 years survivability periods for different BC subtypes. The cohort of their study includes 287 patients from a Spanish region. The patients have received different therapies with sometimes mixed of them (Paredes-Aracil et al., 2017), which makes it difficult to relate the genomic activities to a specific therapy during the survival prediction.

In this present paper, we are extending an earlier supervised learning model that shows preliminary results to predict which BC patients will survive beyond 5 years after undergoing a given treatment therapy (Tabl et al., 2018b). This extended model has been refined and validated by comparison with feature Selection approach mRMD 2.0, visual analysis, and biological validation for set of 12 potential biomarkers (FGF16, ASAP1, FBXO41, FOSB, VAMP4, ARFGAP2, BLP, CT47A1, PRPS1, ICOSLG, ARPC3, ZFP91) from the resulting 47 genes in all classification nodes.

## Materials and Methods

We used a publicly accessible dataset that contains samples for 2,433 breast cancer patients (Curtis et al., 2012; Pereira et al., 2016). The gene expression profiles were totally processed and normalized (Curtis et al., 2012). After studying the given data and selecting only patients who have received one type of treatment, a set of six classes were identified as the base of this work. These classes are the combination of each treatment: surgery (S), hormone therapy (H), and radiotherapy (D) with a patient status as living (L) or deceased (D). The numbers of samples (patients) for each class in the proposed model are shown in Table 1. Data from a total of 347 patients was included in this work.

**Table 1:** List of classes with the number of samples in each class, with the number of genes for each class after filter feature selections.

Class	Number of samples	Number of genes after filter feature selection
Living and Radio (LR)	132	1771
Deceased and Radio (DR)	19	227
Living and Hormone (LH)	20	80
Deceased and Hormone (DH)	6	20
Living and Surgery (LS)	130	1771
Deceased and Surgery (DS)	40	197
Total	347	4066

To avoid overfitting, we performed the filter feature selection first for each class, before running the wrapper feature selection or even the classification model on all the samples from all classes. The number of genes after the filter feature selection for each class are reported in Table 1.

Based on the available data, only three treatment therapies are covered in this study: surgery, hormone therapy, and radiotherapy. Our model uses hierarchical classifiers to classify one-versus-the-rest classes. The classes are imbalanced. Hence, standard classification methods will yield poor performance results. The pipeline starts with feature selection methods like Chi-square (Mantel, 1963) and information gain (IG) that are applied to limit the number of significant features (genes). A wrapper method is also used to obtain the subset of genes that best represents the model by utilizing minimum redundancy maximum relevance (mRMR) (Peng et al., 2005) as a feature selection method. This step is followed by several class balancing techniques, such as the synthetic minority over-sampling technique (SMOTE), resampling, and cost-sensitive to balance the number of classes before applying different types of classifiers, such as naive Bayes (Domingos and Pazzani, 1997) and decision tree (random forest) (Breiman, 2001). Finally, a small number of biomarker genes is recognized for predicting the proper treatment therapy for the patient. To the best of our knowledge, this work is the first prediction model that is built on the combination of the treatment and survivability of the patient as a class.

**FIGURE 1:**
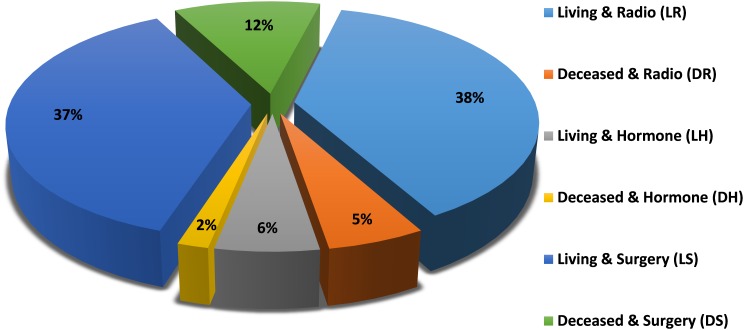
Patient class distribution.

The patient class distribution for the studied model is shown in Figure 1, which shows the percentages of samples within each class. It is clear that there are differences between classes that require class imbalance handling techniques to achieve fair classification.

### Class Imbalance

This model uses a one-versus-rest scheme to tackle the multi-class problem, which leads to an imbalanced class dataset at each node of the classification model. Therefore, we applied the following techniques to handle this issue:

#### Over-Sampling With Synthetic Data

Oversampling the minority class by using synthetic data generators. Several algorithms are used to achieve this. We used one of the most popular ones, SMOTE (Chawla et al., 2002).

#### Using a Cost-Sensitive Classifier

Using penalizing models that apply additional weight to the minority class to achieve class balancing. This, in turn, biases the model to pay more attention to the minority class than others. The algorithm used in this work is called Cost-Sensitive Classifier in Weka machine learning tool using a penalty matrix to overcome the imbalance (Núñez, 1988).

#### Resampling

Replicating the dataset can be using one of two methods: (1) adding copies of the data instances to the minority class, which is called over-sampling (2) deleting some instances of the majority class, which is called under-sampling. We used the over-sampling technique (Gross, 1980).

### Feature Selection

The gene expression dataset contains 24,368 genes for each of the 347 samples. The curse of dimensionality makes it difficult to classify the dataset in its current form. Thus, engaging in feature selection is essential to narrow down the number of genes to a handful at each node. Chi-square and Info-Gain are applied to select the best information gain of the selected genes, this step (Which is usually called filter feature selection) will drop down the number of genes to a couple of hundreds based on the correlation between each class and the gene expressions based on the default correlation threshold in WEKA. After that, mRMR is applied to identify the best subset of significant genes. mRMR (Which is usually considered as a wrapper feature selection) is an algorithm that is commonly used in greedy searches to identify the characteristics of features and correctly narrow down their relevance.

In the trial to find the best feature selection wrapper method to select handful gene biomarker for each class, we applied both mRMD 2.0 and mRMR on the filtered genes on each class. mRMD 2.0 outperformed mRMR fourth and the fifth node as seen in Table 2, while mRMR performed better in the second and third. Both classifiers had 100% of accuracy in the first node, but the lower number of selected genes in that node using mRMR made it more efficient.

**Table 2:** Illustrate the results of using mRMD 2.0 vs mRMR on each node then applying random forest classifier on each node.

Node	mRMD 2.0		mRMR	
	# of Biomarkers	Accuracy	# of Biomarkers	Accuracy
DH VS Rest	20	100.00%	10	100.00%
DR VS Rest	13	99.47%	14	100.00%
LH VS Rest	4	98.25%	9	100.00%
DS VS Rest	13	98.69%	6	97.90%
LR VS LS	10	81.29%	8	80.90%
Total # of Biomarkers	60		47	

### Multi-Class Classification Model

We applied a multi-class approach, the one-versus-rest technique. This approach involves classifying one class against the remaining classes and then removing that class from the dataset. Afterward, we selected another class to classify it against the rest, and so on. Using a greedy method to find the starting node, the method involves classifying all possible combinations, such as DH, against the rest, then DR against the rest, and so on for all six classes. Afterward, the best starting node is selected as the root node for the classification tree based on the best performance.

Several classifiers were tested to achieve these results, including random forest, SVM, and naive Bayes, random forest outperformed the others and has shown a better classification power for the hierarchical model. Therefore, we used it in all nodes. The classification model was built using 10-fold cross-validation. The data is divided into 10 equal folds of samples, then the learning method will loop 10 times, at each time, it will learn from 90 folds and test on the remaining (left out) fold. At each time in the loop, it will take out a unique fold that has not been shown up in the previous loop steps as a left out. The 10-fold modules will increase the learning samples to 90% of the samples, while it will test on 100% of the samples. The sample will be classified around 9 times; the class is voted more will be considered as the predicted class. The accuracy and other performance measurements are calculated based on the testing folds; therefore, the accuracy here is a testing accuracy.

## Results and Discussion

The developed multi-class model also shows the final results for each node and the performance measures that were considered, such as accuracy, sensitivity, F1-measure, and specificity. Moreover, it also shows the number of the correctly and incorrectly classified instances in each node.

**FIGURE 2:**
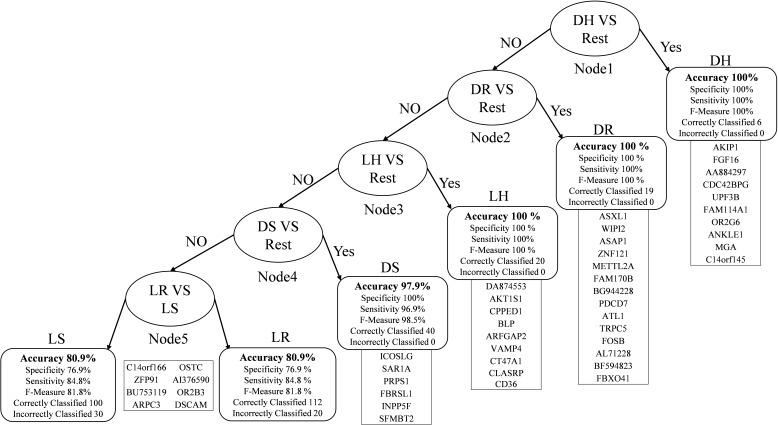
Multi-Class classification model with performance measures.

In Figure 2, the root node is DH against the rest that gives 100% accuracy. The second node is obtained after removing the DH instances from the dataset and then classifying each class against the rest. The best outcome was DR, which had an accuracy level of 100%. We repeated the same technique for the third node, finishing with LH with an accuracy of 100%. Then DS in the fourth node with an accuracy of 97.9%, sensitivity is 96.9%, and specificity is 100% because all the DS samples were correctly classified. In the fifth node, which is the final one, we have LR and LS. The accuracy drops down to 80.9% because it is difficult to distinguish between the living samples in both.

Our method was used to identify the 47 gene biomarkers that are listed in Table 3. Functional validation was conducted and biological Analysis was provided for some genes by studying the information provided in the literature. The genes marked as blue are those that were considered for further biological relevance (see the discussion in the next section).

**Table 3:** Gene biomarkers for each class vs. the Rest at each node.

	DH	DR	LH	DS	LR and LS
Genes	AKIP1	ASXL1	DA874553	ICOSLG	C14orf166
	FGF16	WIPI2	AKT1S1	SAR1A	ZFP91
	AA884297	ASAP1	CPPED1	PRPS1	BU753119
	CDC42BPG	ZNF121	BLP	FBRSL1	ARPC3
	UPF3B	METTL2A	ARFGAP2	INPP5F	OSTC
	FAM114A1	FAM170B	VAMP4	SFMBT2	AI376590
	OR2G6	BG944228	CT47A1		OR2B3
	ANKLE1	PDCD7	CLASRP		DSCAM
	MGA	ATL1	CD36		
	C14orf145	TRPC5			
		FOSB			
		AL71228			
		BF594823			
		FBXO41			

At each node, we tried different standard classifiers to select the classifier with the best accuracy at that node as seen in Table 4, random forest outperformed the other classifiers in all nodes. The accuracy at the difficult node 5 still down compared to the other nodes. However, we can see a significant improvement in this node as it is 80.9% comparing to the second best 77.1% accuracy using SVM with a linear kernel. In node 4, where the accuracy is 97.9% for random forest, the other classifiers performed with very low 79.06% accuracy for the second best which is SVM with radial basis function kernel. Bayesian classifier had the second best performance in the first, second, and third nodes with 99.47, 96.3, and 92.4% accuracies in order. SVM with polynomial degree 3 kernel had an average performance in all nodes compared to the other classifiers.

**Table 4:** Comparison of the standard classifiers at each node of the proposed model.

Node	SVM Linear	SVM Polynomial	SVM RBF	Bayesian Naive Bayes	Random Forest
DH VS. Rest	98.41%	98.68%	97.35%	99.47%	100%
DR VS. Rest	94.46%	95.78%	91.56%	96.3%	100%
LH VS. Rest	89.47%	92.4%	88.3%	92.4%	100%
DS VS. Rest	75.65%	77.23%	79.06%	75.92%	97.9%
LR VS LS	77.1%	74.81%	76.72%	76.34%	80.9%

## Biological Insight

A combination of gene regulation analysis and biological analysis have been done to validate some of the biomarker genes. Biological validation was carried out using relevant literature (Bamberger et al., 1999; Sabe et al., 2009; Tommasi et al., 2009; Dombkowski et al., 2011; Allegra et al., 2012; Caballero et al., 2014; Katoh and Nakagama, 2014; Kechavarzi and Janga, 2014; Nam et al., 2015; Qiu et al., 2015). Figure 4–7 are the circos plots for the relationships between the genes for node 2 and node 3. These plots show the significant coefficient correlation among genes expressions.

**FIGURE 3:**
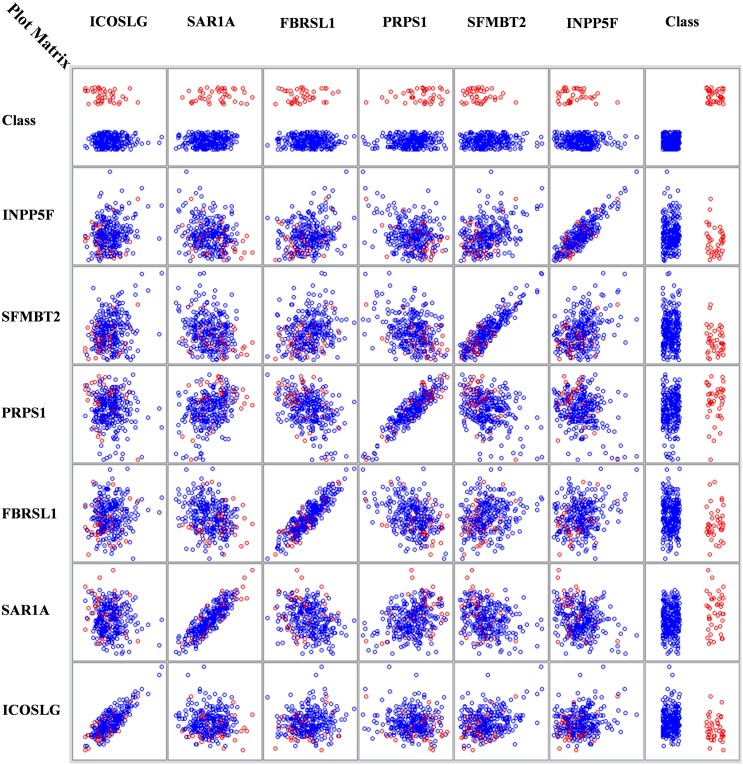
Node Four DS vs. Rest with six genes relations matrix.

Figure 3 is a multi-dimensional representation of the plot matrix for the six biomarker genes found in Node 4 for the DS class vs. the remaining ones, as an example. The figure also shows the relations among the six genes. It is clear from the class column that the samples are separable. The values in *x*-axis represent the gene expression values in the column side, where the *y*-axis represents the gene expression values at the row side.

In the first node, FGF16 gene is a member of the fibroblast growth factors (FGFs) family, which is involved in a variety of cellular processes, such as stemness, proliferation, anti-apoptosis, drug resistance, and angiogenesis (Katoh and Nakagama, 2014). Figure 8 shows that the gene expression of FGF16 is up-regulated and the gene expression of UPF3 is down-regulated in the DH samples compared to the rest of the samples. UPF3 is a regulator of non-sense transcripts homolog B (yeast). Kechavarzi and Janga (2014) found that UPF3 is one of the actively upregulated RNA-binding proteins identified in nine cancers in humans and their cancer relevant references, and breast cancer is one of them.

**FIGURE 4:**
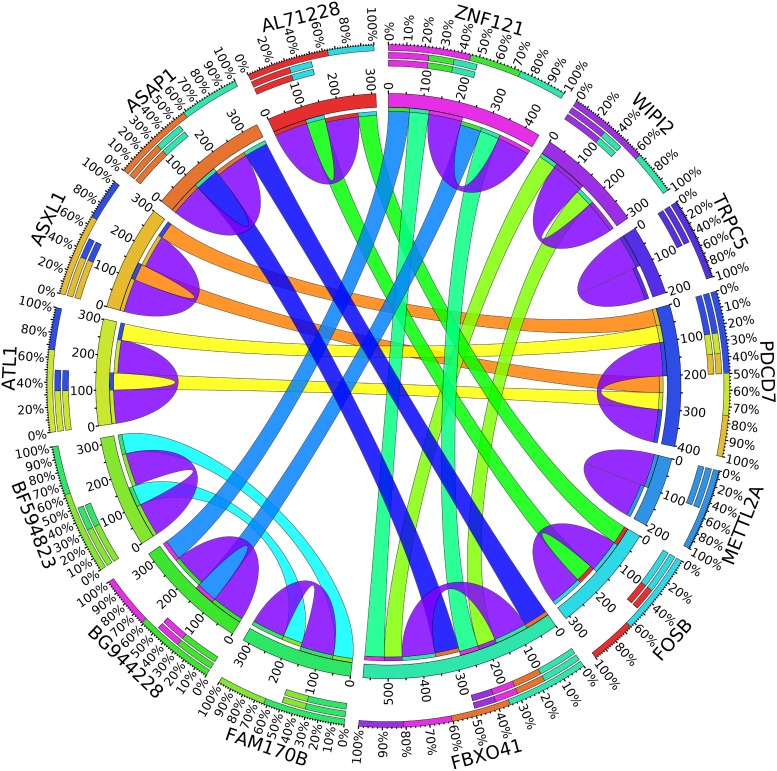
Circos plot for the biomarker genes in node number two for the DR samples based on the correlation coefficient among genes expressions (*p* < 0.05).

**FIGURE 5:**
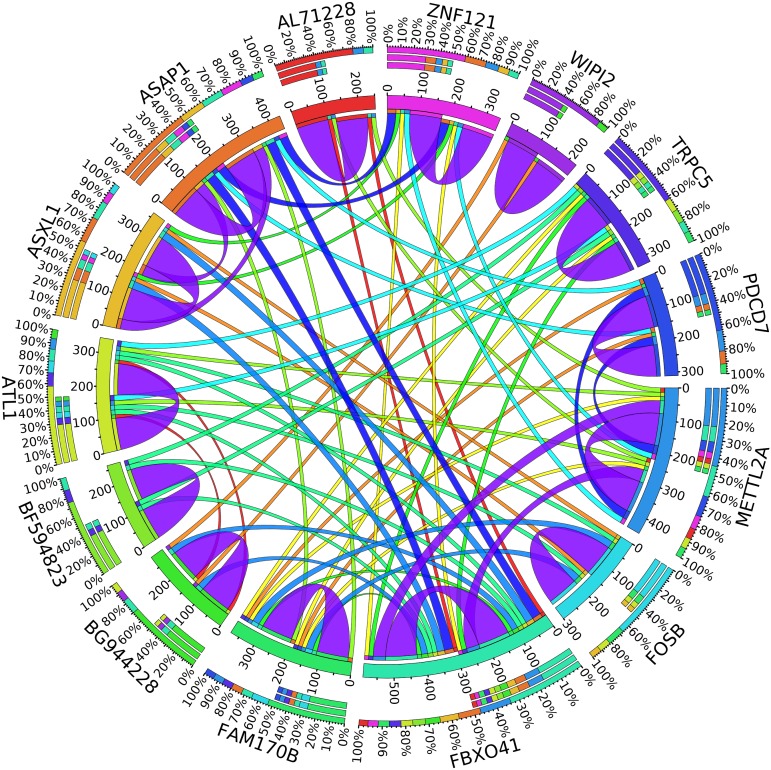
Circos plot for the biomarker genes in node number two for the Rest samples based on the correlation coefficient among genes expressions (*p* < 0.05).

**FIGURE 6:**
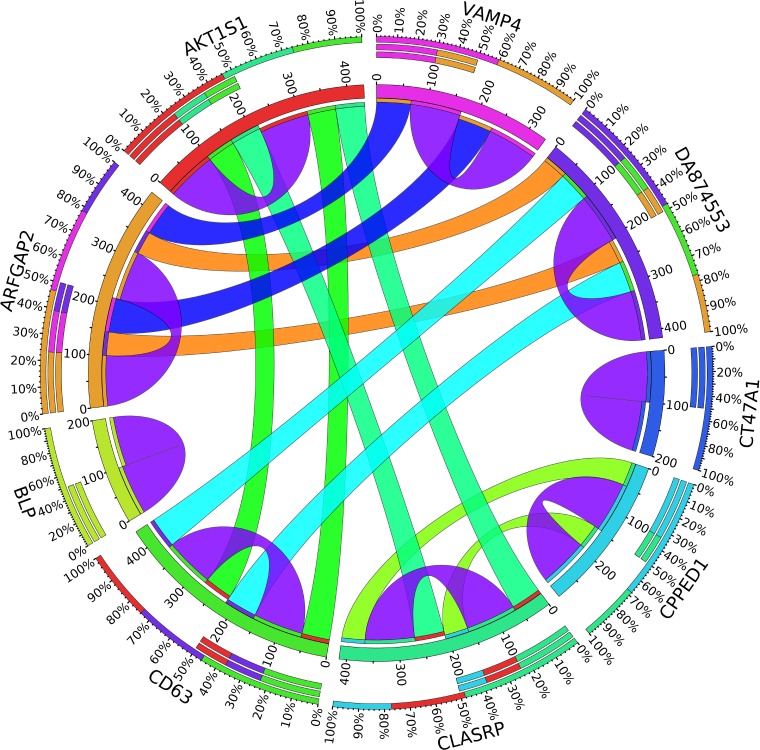
Circos plot for the biomarker genes in node number three for the LH samples based on the correlation coefficient among genes expressions (*p* < 0.05).

**FIGURE 7:**
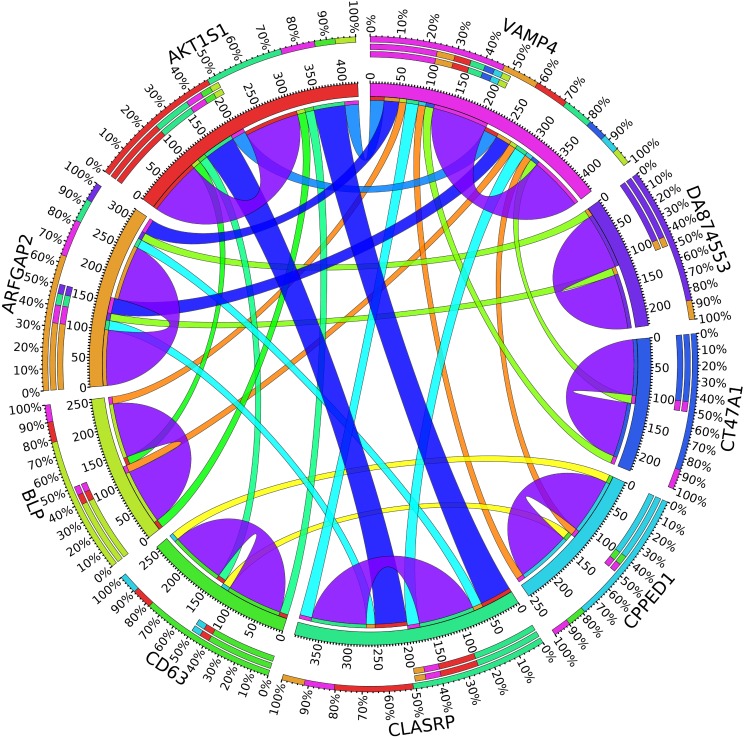
Circos plot for the biomarker genes in node number three for the Rest samples based on the correlation coefficient among genes expressions (*p* < 0.05).

In the second node, ASAP1 is shown to be a breast cancer biomarker; it is precisely correlated to its invasive phenotypes that have not been accurately identified (Sabe et al., 2009). Sabe et al. (2009) reported that ASAP1 is abnormally overexpressed in some breast cancers and used for their invasion and metastasis. As shown in Figure 4, ASAP1 has a strong coefficient correlation with FBXO41 in the DR samples, but it is less correlated with the remaining samples, as shown in Figure 5. Figure 9 shows that the genetic expression of ASAP1 is down-regulated in the DR samples compared to the remaining samples. FOSB is a member of the AP-1 family of transcription factors. Bamberger et al. (1999) concluded that sharp differences in the expression pattern of AP-1 family members are present in breast tumors, and fosB might be involved in the pathogenesis of these tumors (Bamberger et al., 1999). As shown in Figure 6, FOSB has a strong correlation coefficient with AL71228 in the DR samples, but it was not found to be correlated to the remaining samples, as shown in Figure 7.

In the third node, the VAMP4 gene is a target for some cellular and circulating miRNAs in neoplastic diseases, such as miRNA-31. In any case, it has been confirmed that cellular miRNAs are involved in the development of breast cancer(Allegra et al., 2012). As shown in Figure 6, VAMP4 has a strong coefficient correlation with ARFGAP2 in the LH samples, but it is less correlated to the rest of the samples, as shown in Figure 7. Figure 10 shows that the genetic expression of VAMP4 is down-regulated in the LH samples compared to the remaining samples while the gene BLP is up-regulated in the LH samples compared to the remaining samples. CT47A1 is one of seven cancer/testis genes in the CT class. CT genes are significantly overexpressed in ductal carcinoma *in situ* DCIS (Caballero et al., 2014).

In the fourth node, Phosphoribosyl pyrophosphate synthetase 1 (PRPS1) was found to be a direct target of miR124 in breast cancer (Qiu et al., 2015). Nam et al. (2015) stated that ICOSLG is a potential biomarker of trastuzumab resistance in breast cancer, which affects the progression of the disease.

Regarding the fifth node, Dombkowski et al. (2011) studied several pathways in breast cancer. They found that ARPC3 reveals extensive combinatorial interactions that have significant implications for its potential role in breast cancer metastasis and therapeutic development. Zinc finger protein 91 homolog ZFP91 is a methylated target gene in mice. It was identified through methylated-CpG island recovery assay-assisted microarray analysis (Tommasi et al., 2009).

**FIGURE 8:**
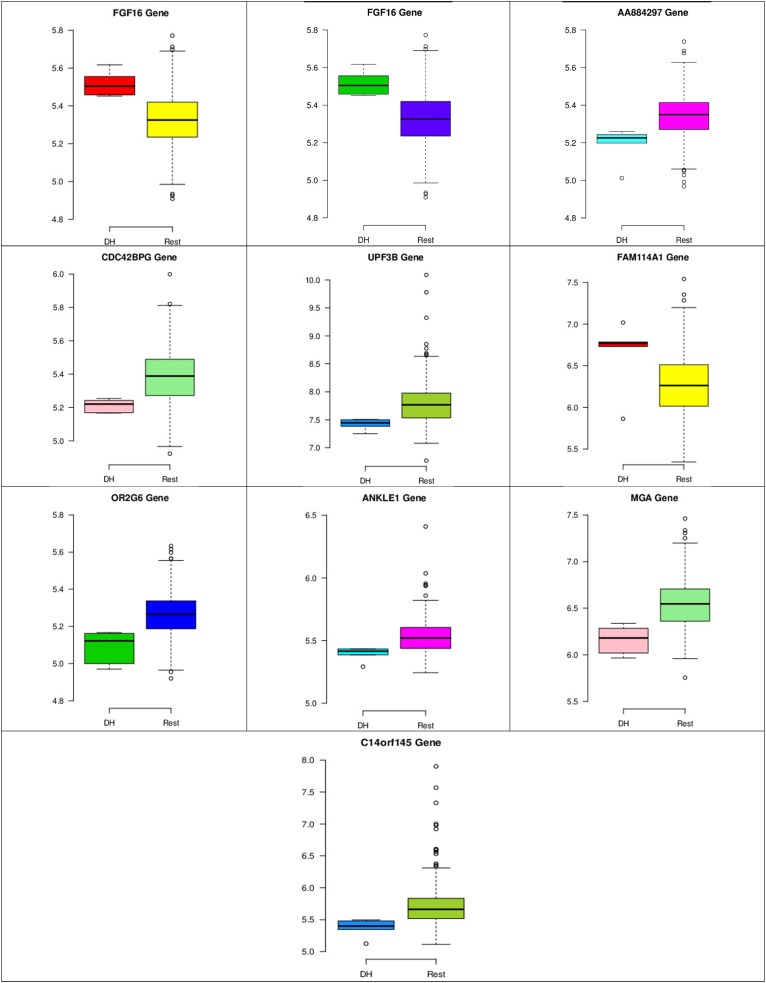
Boxplots for the 10 biomarker genes in node number one show the minimum, first quartile, median, third quartile, and maximum gene expression values for each group of samples (DH vs. Rest).

**FIGURE 9:**
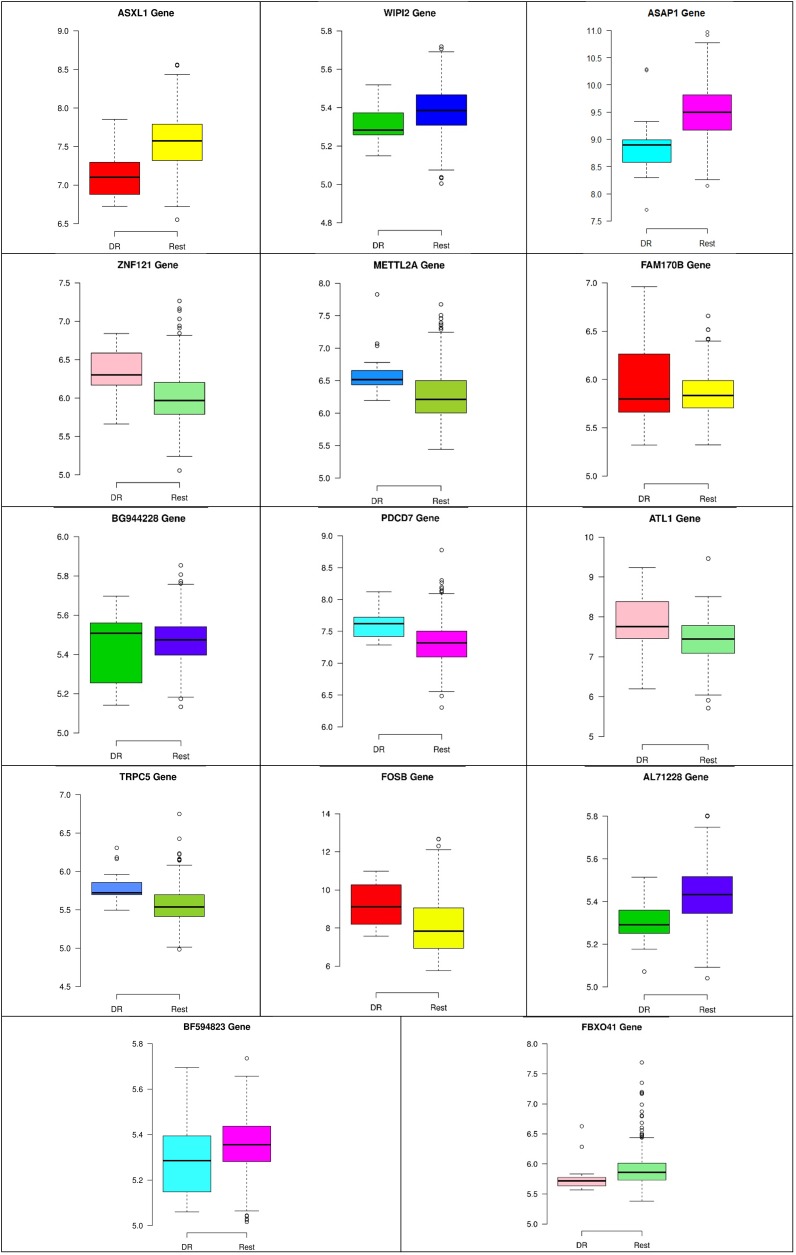
Boxplots for the 14 biomarker genes in node number two show the minimum, first quartile, median, third quartile, and maximum gene expression values for each group of samples (DR vs. Rest).

**FIGURE 10:**
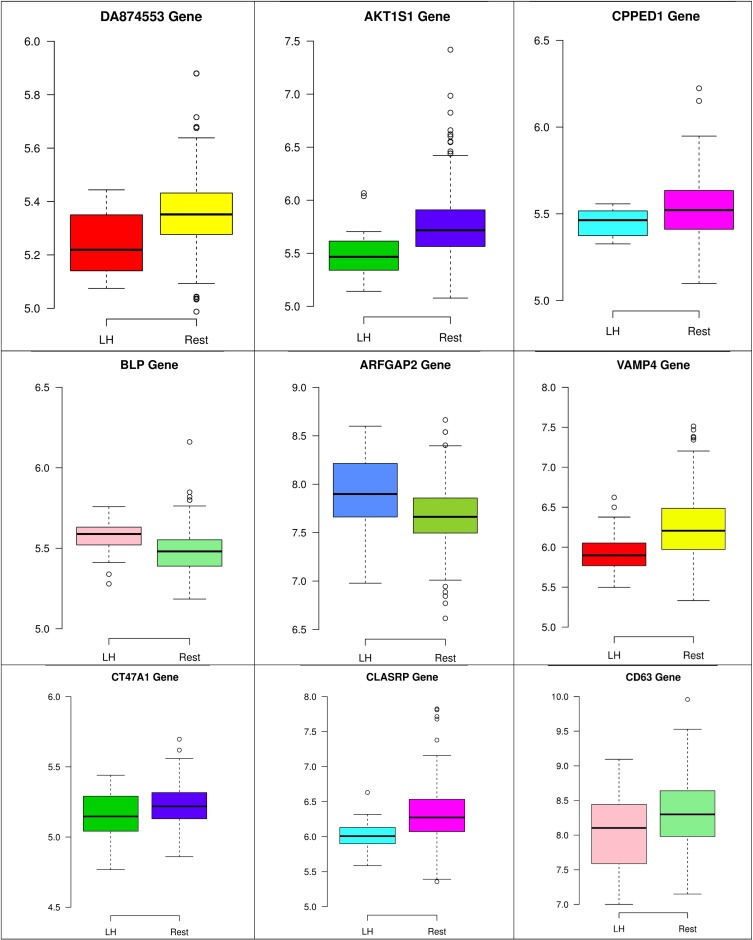
Boxplots for the nine biomarker genes in node number three show the minimum, first quartile, median, third quartile, and maximum gene expression values for each group of samples (LH vs. Rest).

Figure 8–10 show three of the five nodes for each class against the rest boxplot for the gene biomarkers. The plots also show the up-regulated and down-regulated genes. Most of the biomarkers exhibit clear discrimination between the expression values for a specific class sample vs. the remaining samples in the classification node. Many of those biomarkers have outliers, and some of those outliers’ values are in the opposite direction of other class, such as the outliers for the UPF3B gene in the “Rest” class vs. the “DH” class in the first node, as shown in Figure 8. Some others are in the same direction as those of the other class, such as the outliers for the ZNF121 gene in the “Rest” class vs. the “DR” class in the second node, as shown in Figure 9. Some have outliers in both directions, such as the outliers for the ARFGAP2 gene in the “Rest” class vs. the “LH” class in the second node, as shown in Figure 10. The outliers that are in the same direction do not interfere in distinguishing the two classes, even though they may misguide the classifier in other scenarios.

## Conclusion

The use of a machine learning model for identifying gene biomarkers for breast cancer survival is a significant step in determining the proper treatment for each patient and will potentially increase survival rates. This study analyzes the gene activities of the survival vs. deceased for each therapy, and the potential biomarkers will help to identify the best therapy for the patients based on their gene expression test. This model has very high accuracy levels, and it uses a hierarchical model as a tree that includes one-versus-rest classifications.

The computational model pulls sets of biomarkers for patients who received different treatments. These biomarkers can be used to distinguish whether the patient survived or died in a 5-year time window for a specific treatment therapy. Related literature was used to verify the relationships between these biomarkers and breast cancer survivability.

Future work includes testing these gene biomarkers in biomedical labs. This novel model can be improved to be used to identify the proper biomarker genes (signature) for different cancer types or even in cases in which patients need or have received more than one type of therapy. Considering additional patient data will enable researchers to cover all missing treatments. With this considerable data size, big data tools, such as Hadoop and Spark, can be utilized to devise an enhanced model.

## Data Availability

Publicly available datasets were analyzed in this study. This data can be found here: http://www.cbioportal.org/study?id=brca_metabric.

## Author Contributions

AT and AA applied the method. AT retained the results. All authors have equally contributed in brainstorming and writing the manuscript.

## Conflict of Interest Statement

The authors declare that the research was conducted in the absence of any commercial or financial relationships that could be construed as a potential conflict of interest.
